# Newly Emerged Antiviral Strategies for SARS-CoV-2: From Deciphering Viral Protein Structural Function to the Development of Vaccines, Antibodies, and Small Molecules

**DOI:** 10.3390/ijms23116083

**Published:** 2022-05-29

**Authors:** Chunye Zhang, Ming Yang

**Affiliations:** 1 Department of Veterinary Pathobiology, University of Missouri, Columbia, MO 65212, USA; zhangcherryuniversity@gmail.com; 2 Department of Surgery, University of Missouri, Columbia, MO 65211, USA

**Keywords:** COVID-19, SARS-CoV-2, nonstructural proteins, structural proteins, vaccines, antibody treatment, compounds, inhibitors, therapy

## Abstract

Coronavirus disease 2019 (COVID-19) caused by the infection of severe acute respiratory syndrome coronavirus 2 (SARS-CoV-2) has become the most severe health crisis, causing extraordinary economic disruption worldwide. SARS-CoV-2 is a single-stranded RNA-enveloped virus. The process of viral replication and particle packaging is finished in host cells. Viral proteins, including both structural and nonstructural proteins, play important roles in the viral life cycle, which also provides the targets of treatment. Therefore, a better understanding of the structural function of virus proteins is crucial to speed up the development of vaccines and therapeutic strategies. Currently, the structure and function of proteins encoded by the SARS-CoV-2 genome are reviewed by several studies. However, most of them are based on the analysis of SARS-CoV-1 particles, lacking a systematic review update for SARS-CoV-2. Here, we specifically focus on the structure and function of proteins encoded by SARS-CoV-2. Viral proteins that contribute to COVID-19 infection and disease pathogenesis are reviewed according to the most recent research findings. The structure-function correlation of viral proteins provides a fundamental rationale for vaccine development and targeted therapy. Then, current antiviral vaccines are updated, such as inactive viral vaccines and protein-based vaccines and DNA, mRNA, and circular RNA vaccines. A summary of other therapeutic options is also reviewed, including monoclonal antibodies such as a cross-neutralizer antibody, a constructed cobinding antibody, a dual functional monoclonal antibody, an antibody cocktail, and an engineered bispecific antibody, as well as peptide-based inhibitors, chemical compounds, and clustered regularly interspaced short palindromic repeats (CRISPR) exploration. Overall, viral proteins and their functions provide the basis for targeted therapy and vaccine development.

## 1. Introduction of COVID-19

### 1.1. COVID-19 Pandemic

**Figure 1:**
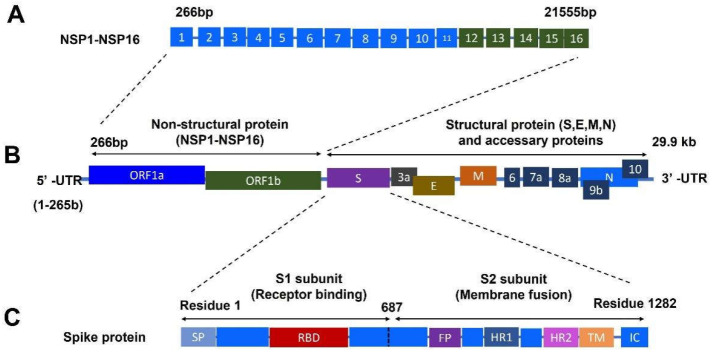
A schematic graphic of the genome-encoded proteins of SARS-CoV-2. (**A**) Genome-encoded nonstructural proteins from NSP1 to NSP16. (**B**) Genome-encoding proteins of SARS-CoV-2 with the structural proteins and nonstructural proteins. (**C**) Full length of S protein of SARS-CoV-2 comprised of subunit 1 (S1) and subunit 2 (S2). Abbreviations: bp: base pair; E: envelop protein; FP: fusion peptide; HR1 and HR2: heptad repeat regions 1 and 2; IC: intracellular tail; kb: kilobase pair; M: membrane protein; NSP: nonstructural protein; N: nucleocapsid protein; ORF1a: open reading frames 1a; ORF1b: open reading frames 1b; RBD: receptor-binding domain; S: spike protein; SP: signal peptide; S1: receptor-binding subunit; S2: membrane fusion subunit; TM: transmembrane; 5′-UTR: 5′-untranslated region; 3′-UTR: 3′-untranslated region.

The COVID-19 pandemic caused by severe acute respiratory syndrome coronavirus 2 (SARS-CoV-2) is a highly contagious disease [1]. The outbreak of COVID-19 led to a global pandemic and resulted in tremendous loss with a high number of deaths (a total of 6,075,512 deaths, 20 March 2022) and infections (469,833,166 total confirmed cases, 20 March 2022) worldwide [2]. The COVID-19 disease causes more severe conditions for the immunocompromised population and accelerates the progression of other diseases [3]. The SARS-CoV-2 virus is a single-stranded enveloped positive-sense RNA virus that belongs to the β-coronavirus [4]. The proteins encoded by the genome of SARS-CoV-2 are comprised of structural (SPs) and nonstructural proteins (NSPs), as well as accessory proteins [5,6]. SPs mainly include spike (S), membrane (M), envelope (E), and nucleocapsid (N) proteins [7]. NSPs contain open reading frames (ORFs), including ORF1a and ORF1b regions. There are 16 NSPs located in the ORF1a and ORF1b regions [5]. A schematic of the genome-encoded proteins of SARS-CoV-2 is summarized in a figure (Figure 1).

In this review, the structure and function of SARS-CoV-2 proteins are updated according to the latest literature reports, including S, M, N, E, and NSP1-NSP16. Here, we mainly focus on the newly deciphered structure and function of SARS-CoV-2 proteins, as they have been predicted or referenced according to the sequence and structure of SARS-CoV-1. Furthermore, we summarize the newly developed anti-SARS-CoV-2 treatment strategies, including antibodies, vaccines, and small molecules.

### 1.2. Structural Proteins of SARS-CoV-2

**Figure 2:**
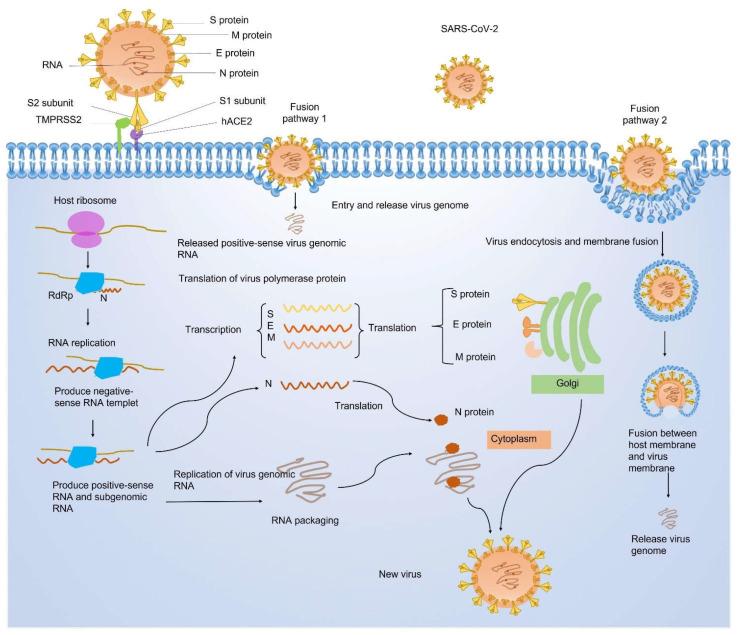
The binding, entry, and package of SARS-CoV-2 in host cells. Genome-encoding proteins are labeled on an enlarged virus. There are two fusion pathways of viral protein with the host cell membrane, including (1) direct fusion with the cell membrane to release the virtual genome RNA and (2) endocytosis via membrane fusion of the viral membrane with a host cell membrane. hACE2: human angiotensin-converting enzyme 2; M: membrane protein; N: nucleocapsid protein; RdRp: RNA-dependent RNA polymerase; SARS-CoV-2: severe acute respiratory syndrome coronavirus-2; S: Spike protein; TMPRSS2: transmembrane serine protease 2.

Spike (S): The SARS-CoV-2 S protein is extensively investigated due to its critical role in the virus entry to host cells. The S protein includes two functional subunits S1 and S2. Subunit S1 plays an important role in viral recognition and binding of the human angiotensin-converting enzyme 2 (hACE2) receptor. Subunit S2 is responsible for the membrane fusion between the host and viral membrane. Those two steps are necessary for SARS-CoV-2 entry into host cells. Thus, the S protein facilitates viral invasion to host cells [8]. SARS-CoV-2 enters host cells through two different fusion pathways [9], either by direct fusion with the cell membrane to release the virtual genome RNA or by endocytosis via membrane fusion of the viral membrane with a host cell membrane. The process of viral entry, infection, and replication is illustrated by a schematic graph (Figure 2). The virus receptor-binding domain (RBD) contains several antigenic epitopes. Those antigenic epitopes, also known as antigenic determinants, are the binding sites of host antibodies. The antigenic epitope plays an important role in activating the host CD4 and CD8 T cell immune response [10]. Therefore, the S protein, the RBD domain, and antigenic epitopes pave the road for the development of vaccines and therapeutic strategies. However, the virus also adjusts itself to increase the ability of invasion and infection. The efficacy of vaccines and therapy is subject to the most frequent mutation.

The deciphering of structural information and functional properties of the S protein plays a significant role in understanding the interactions and underlying mechanisms between the host and virus infection. Recently, researchers discovered that SARS-CoV-2 uses the structural function of the S protein to weaken or escape host immune surveillance. For example, it has been shown that there is a correlation between the high affinity of SARS-CoV-2 variants of concern (VOCs) and the mechanical stability of the S/ACE2 complex, which causes an immune escape and the spreading of the Delta and Omicron variants. The mutations of N501Y (Alpha, Beta, Gamma, and Omicron), E484Q (Beta, Gamma, and Omicron), and Omicron E484A contribute to the increased binding affinity between RBD and ACE2 [11,12]. Additionally, the mechanical stability of the SARS-CoV-2 RBD/S protein seems to protect the virus in the upper respiratory region under mechanical forces compared with that in SARS-CoV-1 [13]. Furthermore, glycosylation is essential for viral virulence since the viral fusion protein is coated with a higher thickness of N-glycan. Mutations of N165A and N234A cause the deletion of glycans, resulting in a weakened binding affinity. Notably, it has been the center of debate in this field on the structural changes (close to open) mediated by N-glycan located on the S protein [14]. Therefore, treatments that control the conformation of the S protein could be a target for vaccine application [15].

Membrane (M): The M protein is one of the structural proteins. It comprises the virus envelope and contributes to viral morphogenesis [7,16]. The similarity of genomic sequences encoding the M proteins between SARS-CoV-1 and SARS-CoV-2 is significantly high [17,18,19]. Thus, the information about the M protein of SARS-CoV-1 provides valuable grounds for further study of the newly emerged SARS-CoV-2. Recently, scientists have applied bioinformatics tools and found detailed structure information of the M protein in SARS-CoV-2. Although there are some differences in amino acids between SARS-CoV-2 and SARS-CoV-1, they possess a similar protein-structure property. For example, both M proteins display hydrophobic features in the transmembrane domains and hydrophilic features in N-terminals and C-terminals. The traits of hydrophilicity and hydrophobicity are both critical for vaccine development. In the same study, the authors also found potential B-cell epitopes that may be associated with the recognition of antibodies from the host immune response. These epitopes have the potential to be applied for further vaccine development or treatment targets [20]. Another group using in silico analyses found a transmembrane domain of the M protein in SARS-CoV-2 with three helixes, which forms a sugar transporter-like structure, in terms of semiSWEET. The structure of semiSWEET indicates that the M protein may play multifunctional roles, such as sugar transport and metabolism. This interesting finding inspires a further investigation into the function of the M protein [21].

Envelope (E): The E protein plays an essential role not only in viral morphogenesis as an ion transporter but also in serving as a virulence factor. The envelope protein of SARS-CoV-2 shows the function of E-mediated virulence [22]. A study demonstrated an interaction between the viral E protein and human lung epithelium tight junction protein PALS1 (protein associated with Lin-seven 1) or MPP5 (membrane-associated palmitoylated protein 5). In the study, the researchers provided structure-based evidence on the interaction between the E and PALS1 proteins [23]. This structure discovery further explained the underlying mechanism of biological phenomena. It supports the evidence that a high level of E-protein was expressed in the SARS-CoV-2-infected lung tissues [24]. Another well-designed study demonstrated the E protein virulence factor could modulate the immune response by suppressing and inducing the Nod-like receptor protein 3 (NLRP3) inflammasome at different stages of the infection. The authors highlighted the importance of targeting the SARS-Cov-2 E protein for the development of vaccines or therapeutic strategies [25,26]. By cooperating with the analysis of the viral structure and infection ability, we can better understand the underlying mechanism of COVID-19 infection and find a precise target for treatment. 

Nucleocapsid (N): The N protein, in which the viral genome is encapsulated, plays a fundamental function in viral RNA transcription, replication, and virion assembly [27]. Several groups reported that inhibiting RNA-induced phase separation of the N protein by bioactive molecules can interrupt the SARS-CoV-2 replication cycle [28,29,30,31]. The N protein can inhibit the phosphorylation of the signal transducer and activator of transcription 1 (STAT1) and STAT2, which results in the inactivation of the host interferon (IFN) signaling pathway. Consequently, the viruses can escape from the host IFN-mediated antiviral response [32]. An in vitro study showed that the N protein can induce the phosphorylation of nucleic factor (NF)-ĸB p65 and modulate the production of proinflammatory cytokines and the M1 polarization of macrophages. The mentioned results were further confirmed by denatured N-protein and N-protein antibody treatment. In a mouse model, N protein-mediated activation of NF-ĸB signaling pathway induced acute lung injury [33]. Therefore, the N protein is also an attractive target for exploring treatment for COVID-19 [27,34].

### 1.3. Nonstructural Proteins of SARS-CoV-2

NSP1: The NSP1 of SARS-CoV-2 has been reported to play a pivotal role in suppressing the translation of host mRNA. Meanwhile, NSP1 facilitates the translation of viral mRNA. In other words, NSP1 can disable the host mRNA translation and shift the process to viral mRNA translation in infected cells. NSP1 shifts the function of host ribosome in a bipartite mechanism. First, the C-terminal domain of viral NSP1 can directly bind with the 40S ribosomal subunit to block the binding of host mRNAs, resulting in the suppression of host mRNA translation. Second, NSP1 can release this inhibition by interacting with a 5′ untranslated region (5′ UTR) of SARS-CoV-2 mRNA with the N-terminal domain of NSP1. Thus, viral mRNA can be translated by the host ribosome [35]. Therefore, viral NSP1 plays an essential role in converting the infected cell from the location for host mRNA translation into a location for viral mRNA translation. This finding triggers the interest in exploring the potential therapeutic options by targeting NSP1.

NSP2: NSP2 has been found to be associated with the Ca^2+^ exchange in the endoplasmic reticulum (ER) and involved in the mitochondria biogenesis [36]. A recent study revealed that the NSP2 of SARS-CoV-2 contains a conserved zinc-binding site that is associated with the binding of RNA. The structural analysis also revealed that the protein surface of NSP2 evolves fast, which may be potentially associated with the interaction of NSP2 and the host [37]. A series of studies and analyses also uncovered that NSP2 may contribute to the interaction between virus and host, such as the interactions with endosomes, ribosomal RNAs, and modulators of translation [37]. Although more investigation is needed to further evaluate the function of NSP2, current studies shed light on the potential of using NSP2 as one of the therapeutic targets. 

NSP3: NSP3, also known as papain-like protease, is responsible for the cleavage of viral polypeptides NSP1-4 [37]. Recently, a new function of the NSP3 of SARS-CoV-2 was reported. The study showed that NSP3 also can directly cleave the interferon regulatory transcription factor 3 (IRF3), resulting in a blunted type I IFN response [38]. As a result, SARS-CoV-2 infection causes the interruption of the host antiviral response. Another study also revealed the interaction between the NSP3/papain-like protease (PLpro) and host antiviral proteins such as IFN-associated proteins [39]. An in silico study showed the molecular mechanism of the interaction between NSP3 and the C-terminal domain of viral N phosphoprotein. The major residues involved in the biding site were analyzed using the molecular docking method [40]. The above-mentioned studies provide inspiring insight into the potential of using NSP3 as a target for therapeutic development, including vaccines and NSP3 inhibitors.

NSP4: Except for its crucial function in viral replication, a recent study revealed that NSP4 plays a role in ER homeostasis. In this study, the highly enriched NSP4 and NSP2 of SARS-CoV-2 were detected in mitochondria-associated ER membranes (MAM). The authors also found that there are shared NSP4 interactors for different viruses (SARS-CoV-1, SARS-CoV-2, and hCoV-OC43), such as N-linked glycosylation machinery and unfolded protein response (UPR)-associated factors, and unique NSP4 interactors for SARS-CoV-2, such as the monoubiquitin–ribosomal fusion protein (RPS27A), a Golgi/ER-resident zinc receptor, and necroptosis (SLC39A7) and the ER-resident Hsp70 chaperone BiP (HSPA5) [36]. ER homeostasis is essential for host cell survival. The disruption of ER homeostasis caused by viral infection could result in ER stress and cell dysfunction, which is essential for viral replication and maturation [41]. 

NSP5: NSP5, also known as 3C-like protease (3CLpro), is found to play an important role in affecting host immune response. Similar to NSP3, NSP5 can also directly cleave IRF3 to block type I IFN response. Meanwhile, NSP5 can cleave the NLR family pyrin domain containing 12 (NLRP12) and TAK1-binding proteins (TAB) to enhance the production of inflammatory cytokines and influence the downstream signaling pathway [38]. A remarkable discovery identified the distinct mechanism of NSP5 on the host immune signaling pathway. NSP5 directly cleaves the amino acids of retinoic acid-inducible gene I (RIG-I) and consequently disables its ability to activate the mitochondrial antiviral signaling (MAVS). Additionally, NSP5 also plays the function of promoting the degradation of MAVS. The study also showed that NSP5 can interact with RIG-I or MAVS to suppress antiviral immune responses, such as inhibition of IFN expression [42]. In a separate study, scientists found that NSP5 caused a significantly increased expression of cytokines such as interleukin (IL)-1β, IL-6, IL-2, and tumor necrosis factor alpha (TNF-α) in a non-small-cell lung cancer cell line Calu-3 and a monocyte cell line THP1. The molecular mechanism study further showed that NSP5 increased the protein level of MAVS and led to activation of the nucleic factor (NF)-κB signal pathway by regulating the SUMOylation of MAVS. The NSP5-mediated activation of the NF-κB signaling pathway can be attenuated by the knockdown of MAVS or the inhibition of SUMOylation [43]. In summary, NSP5 is an attractive target because of its association with the host immune response and cytokine storm.

NSP6: A study revealed that SARS-CoV-2 NSP6 impeded autophagy function in lung epithelial cells. Autophagy is the protective mechanism for host cells to suppress inflammasome activation. NSP6 can directly bind with ATP6AP1 (ATPase H^+^ transporting accessory protein 1) and trigger the inflammasome to induce pyroptosis, which results in severe inflammatory cell death. The NSP6 L37F point mutation decreased the binding affinity with ATP6AP1, causing less damage to virus-infected cells. In addition, the epidemiology analysis showed the NSP6 L37F mutation was associated with asymptomatic COVID-19 infection in human patients. In a word, NSP6 can be further investigated as one of the important therapeutic targets, especially from the perspective of inflammation [18,44,45]. 

NSP7, NSP8, and NSP12: Most interestingly, scientists successfully obtained the Cryo-EM structure of a core polymerase complex comprising the NSP7, NSP8, and NSP12 of SARS-CoV-2. This complex is well-known as the RNA-dependent RNA polymerase (RdRp) complex, which provides structure-based evidence of the interaction among these three proteins. In this polymerase complex, NSP7 and NSP8 serve as cofactors, while NSP12 serves as a catalytic subunit. The study also revealed that there is a high identity of sequence structure of NSP7 and NSP8 compared with the counterpart of bat coronavirus RaTG13; however, there are four residue differences in NSP12 between SARS-CoV-2 and bat coronavirus RaTG13. Meanwhile, the study showed the thermostability of the polymerase subunit was lower for SARS-CoV-2 than that in bat coronavirus. This observed divergence of viral polymerase thermostability between humans and bats could be explained from the perspective of virus evolution. Viruses adapt to human body temperature by reducing their thermostability for viral replication due to body temperature differences between humans and bats [46].

NSP9: The biochemical study of SARS-CoV-2 demonstrated that the nucleoside monophosphate (NMP)ylation of NSP9 is essential for virus replication. The N-terminus of NSP9 NMPylation was catalyzed and mediated by the NiRAN (nidovirus RdRp-associated nucleotidyltransferase) domain of NSP12. Mutational studies were carried out to identify the critical residues of NiRAN-mediated NSP9 NMPylation and virus replication. Results showed that NSP9 N3826 residue plays an important role in the interaction between NSP9 and NSP12, as well as viral replication, which provides insight into the possibility of a drug targeting site [47].

NSP10–NSP14 complex: NSP14 is guanine-N7 methyltransferase (ExoN). Nsp10 coupled with NSP14 forms the NSP10–NSP14 complex. A well-designed study elucidated that the NSP10–NSP14 complex acts as the function of RNase. The RNase activity was improved by the presence of the RNA polymerase complex (a complex of NSP12–NSP7 and NSP8). NSP8 played a significant role in enhancing the activity of the NSP10–NSP14 complex. In addition, the study also showed that the NSP10–NSP14 complex contributed to the repairment of the RNA replication, except for the classic proof-reading function. When RNA replication was stalled due to unpredictable reasons, the NSP10–14 complex can mediate the repair process with RNase activity to maintain the continuity of the elongations [48]. Another study found that NSP14 has antagonizing properties to IFN [49]. Therefore, these discoveries also provide a rationale for targeting these NSPs.

NSP10–NSP16 complex: NSP16 is a RNA methyltransferase. The complex of NSP10–NSP16 plays a critical function in the methylation of viral RNA at the 2′-O position of ribose. This methylation led to the change in the viral RNA cap into a structure that imitates host cellular mRNAs. Using this mechanism, SARS-CoV-2 could evade the host innate immune system [50]. The structure analysis demonstrated the attractiveness of targeting this protein complex as an antiviral strategy by designing an inhibitor to block nucleotide-binding sites [51].

NSP11: Notably, NSP11 is a very short peptide for SARS-CoV-2, which is comprised of only 13 amino acids. A molecular dynamics simulation study identified that NSP11 acts as an intrinsically disordered protein. The conformation change in NSP11 from disorder to order is dependent on the membrane environments. This finding indicates that NSP11 may contribute to the interaction between the virus and host cell membrane. It is worthy of being further investigated due to its important role at the cellular level [52]. 

NSP12: NSP12 is also known as a RdRp. In addition to forming a complex with NSP7 and NSP8, NSP12 was shown to be associated with the receptor-interacting serine/threonine-protein kinase 1 (RIPK1)-signaling pathway. Cytokine storm is the major cause of death in COVID-19 patients. RIPK1, a well-known inflammation mediator, contributes to inflammation and cell death. Activation of RIPK1 was found in human patients with COVID-19 infection. The inhibition of RIPK1 by inhibitors can decrease the viral load in cultured lung organoids from human COVID-19 patients. In addition, the administration of a therapeutic dosage of RIPK1 inhibitors decreased the mortality of virus-infected ACE2-transgenic mice, as well as reduced viral load. They further discovered that NSP12 is responsible for RIPK1 activation, as amino acid variations of NSP12 resulted in different levels of activation of the RIPK1 signaling pathway [53]. These results illustrate the important association between NSP12 and the RIPK1 inflammatory signaling pathway. Thus, targeting NSP12 is an approach for treatment.

NSP13: NSP13 is a helicase protein. It is responsible for virus genome replication. Cryo-EM structure revealed that NSP13, together with NSP7, NSP8, and NSP12, forms a unique complex. A zinc-binding domain is located at the N-terminal of NSP13, while the helicase domain is located at the C-terminal. Together, they confer potent enzymatic functions, such as unwinding RNA/DNA helicase (5′-3′), RNA 5′-triphosphatase, NTPase, and dNTPase. Given the multifunctional and essential roles of this complex, NSP13 is highly conserved between SARS-CoV-1 and SARS-CoV-2, with only one single amino acid variation at position 570 (Ile for the SARS-CoV-1 and Val for SARS-CoV-2). It has been found that NSP13 possesses a significant interferon-antagonizing feature [49]. Thereby, NSP13 is one of the promising drug targets for virus therapeutic strategies [54,55].

NSP15: The endoribonuclease of coronavirus is encoded by NSP15. Previous studies on SARS-CoV-1 discovered that NSP15-encoded endoribonuclease can cleave the virus 5′-polyuridines RNA and facilitate the virus escaping from host macrophage detection [56,57]. The NSP15 of SARS-CoV-2 also showed a strong interferon-antagonizing feature [49].

In summary, both the SPs and NSPs of SARS-CoV-2 play essential roles in viral infection, COVID-19 pathogenesis, and host immune interaction. Uncovering the structure-function properties of the virus and infection-associated underlying mechanisms is essential for treatment development.

### 1.4. Accessory Proteins

Accessory proteins are located within the structure region and contain ORF3a, ORF3b, ORF6, ORF7a, ORF7b, ORF8, ORF9b, ORF14, and ORF10. Both ORF3a and ORF6 were identified to significantly contribute to the pathogenesis of the SARS-COV-2 virus in the hACE2 transgenic mouse model [58]. This study provides an insight into the exploration of attenuated virtual vaccine development. More important studies have revealed the interesting and valuable properties of accessory proteins [59,60]. Here, we do not further discuss them in this paper.

## 2. Vaccine Approach to Prevent Infection

A vaccine is one of the most effective preventative strategies to prevent highly contagious infections, including COVID-19 infection. Here, we discuss strategies to develop vaccines based on viral components.

### 2.1. SARS-CoV-2 Inactivated Vaccines

A viral vaccine is a vaccine that targets the whole virion, including the live-attenuated virus vaccine and inactive virus vaccine; both require the handling of viral infection agents for the purification of viruses to produce vaccines [61]. Live-attenuated vaccines have been successfully applied to protect against various diseases. However, the major disadvantage of their use is the potential reversion to gain virulence and become a disease-causing factor [62]. In contrast, an inactivated vaccine does not have the ability to revert to an unwanted state [63]. The whole inactivated SARS-CoV-2 virion that was generated in Vero cells has been used as an inactivated vaccine. The approved inactivated vaccines include the Sinopharm vaccine produced by the Beijing Institute of Biological Products Co., Ltd. (BIBP) (Beijing, China) [64,65], the Sinovac COVID-19 vaccine produced by Sinovac Life Sciences Co., Ltd. (Beijing, China) [66], and the COVAXIN vaccine developed by Bharat Biotech [67,68]. Vero cells have been applied to develop these vaccines. Other inactivated vaccines, such as the CovIran^®^ vaccines investigated by Shifa Pharmed–Barkat, are waiting for clinical evaluation. The IMBCAMS vaccine is still under development [69]. There are many benefits of using inactivation technology for vaccine development as a traditional tool [70,71]. However, the development and production process of inactivated vaccines need more time due to the requirement for virus culture and inactivation procedure [72].

### 2.2. Protein-Based Vaccines

**Figure 3:**
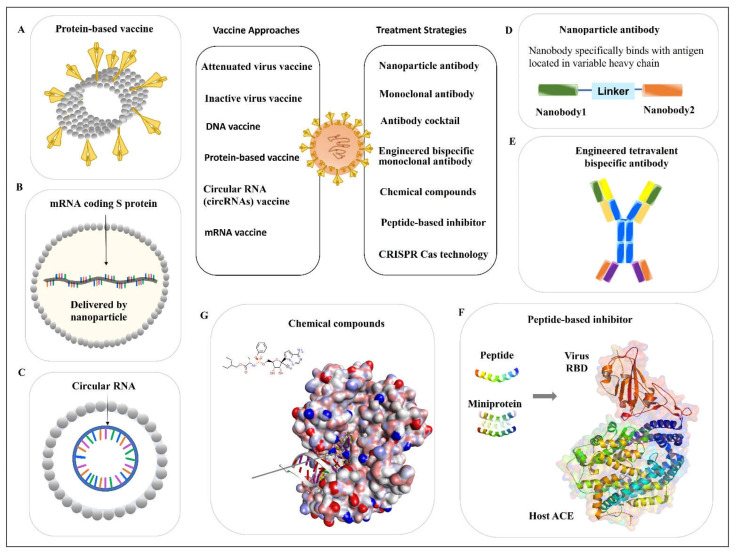
Vaccines and pharmaceutical strategies against SARS-CoV-2 infection. The treatment options for COVID-19 infection are shown in cartoons. Current main treatment options against SARS-CoV-2 infection include (**A**) protein-based vaccines, (**B**) mRNA vaccines, (**C**) circular RNA vaccines, (**D**) nanoparticle antibodies, (**E**) engineered tetravalent bispecific antibodies, (**F**) peptide-based inhibitors (Protein Data Bank/PDB: 6M0J), and (**G**) chemical compounds (PDB: 7BV2). Abbreviations: ACE2: angiotensin-converting enzyme 2; CRISPR/Cas: clustered regularly interspaced short palindromic repeats–associated nucleases; RBD: receptor-binding domain.

Protein-based vaccines do not require handling of the live and attenuated viruses. These kinds of vaccines are stable and easy to distribute. NVX-CoV2373, developed by NOVAVAX, belongs to protein-based vaccines. The vaccine is composed of a nanoparticle core, around which is the recombinant protein antigen of SARS-CoV-2, the sequence of spike (S) protein from the strain Wuhan-Hu-1 (Figure 3A). The vaccine nanoparticles are then mixed with Matrix-M™ adjuvant to produce the ready-to-use vaccine products. An immunology study showed that this vaccine can provoke host protection by inducing robust immune responses, including both cellular and humoral immunity [73]. A clinical trial showed the protection efficacy was 90% against multiple mutations such as variants Alfa, Beta, and Delta. This vaccine has been approved for emergency use for the prevention of COVID-19 in many countries [74]. Other protein subunit-based vaccines either are under investigation or in ongoing clinical trials.

### 2.3. Nucleic Acid-Based Vaccines

#### 2.3.1. DNA Vaccine

The nucleic-acid-based vaccines include DNA vaccines (e.g., DNA plasmids) and RNA vaccines. DNA vaccines do not contain any infectious pathogens. This is one of the safety advantages compared with weakened viral vaccines. DNA vaccines also have an advantage in stability compared with RNA vaccines [75]. Since the outbreak of COVID-19, different DNA-based vaccines have been explored and some of them put into clinical trials. According to the World Health Organization [69], most current DNA vaccines for SARS-CoV-2 are encoded for the S protein. However, there are differences in the vectors. For example, adenovirus ChAdOx1 is used as the vector for the AZD1222 Vaxzevria vaccine [76]. The adenovirus type 26 (Ad26) is utilized as a vector for the Janssen–Cilag International NV (Belgium) Ad26.COV2.S COVID-19 vaccine [77]. Human adenovirus has been applied as a vector for the development of the Sputnik V vaccine [78]. These aforementioned DNA vaccines have been approved for emergency use for the prevention of COVID-19. The adenovirus Type 5 Vector has also been used for the development of the Ad5-nCoV vaccine, which is under clinical trial evaluation [69]. 

#### 2.3.2. mRNA Vaccine

The mRNA vaccine has received considerable attention as a powerful tool to combat against the COVID-19 pandemic. Compared with the traditional vaccines, mRNA vaccines have irreplaceable advantages. For instance, mRNAs are faster and easier to produce. As soon as the virus sequence is decoded, the development of mRNA vaccines can be initiated in a time-saving fashion. There is no need to handle highly infectious pathogens, such as viral isolation and culture. However, some challenges for the development of mRNA vaccines also exist. The mRNA is not as stable as the DNA vaccine and is extremely sensitive to high temperatures, which places difficulty on vaccine distribution. The shortness of the half-life is the innate feature of mRNAs. Thus, the nucleotide needs to be modified to enhance its stability. The uridine modifications to reduce the unfavored immunogenicity to hosts also need to be considered for mRNA vaccine development [79].

Upon the outbreak of COVID-19, two mRNA vaccines were developed and broadly used for disease protection, including BNT162b2 from BioNTech/Pfizer [80] and mRNA-1273 from Moderna [81]. Both vaccines are nucleoside-modified mRNAs encapsulated by lipid nanoparticles as delivery vectors (Figure 3B). They are designed based on stabilized prefusion of the SARS-CoV-2 S protein (the full-length S protein). Clinical trials results show the protection efficacies of BNT162b2 (ClinicalTrials.gov, NCT04368728) [80] and mRNA-1273 (ClinicalTrials.gov, NCT04470427) [81] against SARS-CoV-2 infection were 95% and 94.1%, respectively.

#### 2.3.3. Circular RNA (circRNA) Vaccine

The circRNAs (Figure 3C) have several advanced properties compared with the linear mRNAs. For example, compared with the liner mRNAs, the circRNAs are not sensitive to the exonuclease-mediated degradation due to the lack of 5′ or 3′ ends. Additionally, circRNAs are more stable and have a relatively longer half-life compared with linear mRNAs [82,83,84]. The circRNAs are less likely to induce undesired immunogenicity by escaping the detection from host Toll-like receptors (TLRs). In addition, the expression duration of circRNA-translated proteins is extended due to their resistance to RNases. Similarly, the induction of less immunogenicity has also been observed from uridine-modified mRNAs. However, it is important to notice that uridine modification is needed for mRNAs to avoid detection by TLRs, but no modification is required for circRNAs [85]. The most recent study reported that a novel circRNA vaccine showed robust protection against SARS-CoV-2 infection in a mouse model and rhesus macaques. In this study, the researchers also found the circRNARBD-Delta vaccine showed a protective effect not only from the Delta mutation but also against the Omicron variant, which provides an advantage over the circRNARBD-Omicron vaccine that is only effective on the Omicron variant [86]. These results provide an optimistic insight into further investigation of the circRNA vaccine against virus infection. The circRNA vaccines hold great promise for the future.

The success of vaccination application also highly depends on vaccine type and viral variants. Moreover, a high-efficacy route of vaccine delivery and ease of administration should also be prioritized for vaccination. It has been proposed that the nasal vaccine holds promise for the next generation of anti-respiratory virus vaccines since it is close to the natural infection route [87,88,89]. Several studies have demonstrated that there are many advantages of using nasal or intranasal as a vaccine delivery path. For example, nasal spray vaccines have the advantage of ease of administration compared with intramuscular shots. This provides the convenience of use for both kids and adults. An intranasal vaccine for COVID-19 showed a higher level of mucosal immunity and a low level of viral load in upper and lower respiratory tracks compared with the intramuscular vaccination in a mouse model [90]. Intranasally administrating vaccines generated with the recombinant adenovirus type-5 expressing SARS-CoV-2 N protein to BALB/c mice induced immune responses both locally and systemically [91].

The big challenge for the RNA vaccine is virus mutation, which is applied to escape host surveillance. To overcome this problem, uncovering the most conserved functional RNA sequence and its structure and function is critically important. In addition, the pathogenesis of viral infection disease needs to be well-investigated, including the processes of viral infection, invasion, and replication and the interaction between the virus and host. Based on these aspects to select a target sequence for vaccine development, RNA vaccines will be less subject to the virus mutation.

## 3. Antiviral Antibodies

Therapeutic antibodies for SAR-CoV-2 include a monoclonal antibody (mAb) and a cocktail of several monoclonal antibodies. Some antibodies are developed to block the binding site of the virus to the host. Others are developed for inflammatory modulation.

### 3.1. Monoclonal Antibody

#### 3.1.1. Monoclonal Antibody from Human Patients

Bebtelovimab (LY-CoV1404) is a mAb that neutralizes the SARS-CoV-2 spike glycoprotein RBD. It is a human immunoglobulin G-1 (IgG1) mAb. In vitro studies showed that it retained the binding to S proteins with a broad range of virus variants. Clinical evaluation on the effect of Bebtelovimab alone or together with other monoclonal antibodies Bamlanivimab and Etesevimab is ongoing [92,93]. It has been recommended as an alternative option for managing COVID-19 only when the preferred small molecules Ritonavir-boosted nirmatrelvir and Remdesivir are not available.

#### 3.1.2. Nanoparticle Antibody

Recently, Wu et al. characterized some RBD-specific neutralizing nanobodies and selected a nanobody library derived from alpaca immunization with the SARS-CoV-2 spike glycoprotein. The identified nanobodies showed a neutralizing function against the wild-type and Delta variants of SARS-CoV-2, which are specifically bound with a virus antigen [94]. Then, they also engineered a combined nanoparticle antibody using two nanobodies and a linker (Figure 3D), which showed a significantly increased neutralizing effect compared with every single particle [94]. These findings highlight the possibility of exploring the antibody from different sources and using multiple nanobodies.

#### 3.1.3. Cross-Neutralizer Antibody (Across SARS-CoV-2 and SARS-CoV-1)

This is an exploration of a cross-neutralizer antibody that across the coronavirus family may provide an optimal option. This approach may lead to a more precise binding target to avoid mutation-caused antibody inefficacy. Because the cross-neutralizer requires the antibody to bind with the most conserved viral region, it is less subject to frequent virus mutation. Thus, the neutralization efficacy could be maintained regardless of the mutation. For instance, an in vitro study showed that human mAb 47D11, which bound the core conserved epitope of virus RBD, displayed cross-neutralization for both SARS-CoV-2 and SARS-CoV-1 [95]. This result encourages the further investigation of using 47D11 as the cross-neutralizer for the treatment of COVID-19, either alone or in combination with other treatment options. Cross-normalization antibodies can cope with the weakened binding affinity induced by virus mutation, which is an optional direction for antibody development against viral infection.

#### 3.1.4. Constructed Cobinding Antibody Using Paired or Multiple IgG Sequences

The constructed cobinding antibody has been developed by constructing paired or multiple sequences of a single antibody. It is an approach distinct from an antibody cocktail. The cocktail is made up of mixing different antibodies for treatment. The resulting function relies on the synergistic neutralizing effects that are contributed by different antibodies. In contrast, the cobinding-constructed antibody is made by expressing the sequences of two antibodies in a cobinding manner. For example, by cloning human monoclonal antibodies from recovered human COVID-19 patients, Chen et al. attained three pairs of IgG variable heavy chains (VHs) and light chains (VLs) from memory B cells. Using the paired IgG VH and VL sequences, they developed three monoclonal antibodies. Two of them, namely 311mab-31B5 and 311mab-32D4, showed an effectively neutralizing effect against SARS-CoV-2, evidenced by enzyme-linked immunosorbent assay (ELISA) and flow cytometry-based blockade assays [96]. These antibodies can block the interaction between the virus binding domain and human cell receptor (RBD-hACE2) [96,97]. These findings provide a fundamental basis for exploring a new treatment strategy.

#### 3.1.5. Dual Functional Monoclonal Antibody

One study explored the dual functional mAbs targeting S glycoprotein, consisting of VIR-7831 and VIR-7832. They were derived from a parent antibody (S309) isolated from memory B cells of a patient who survived the 2003 SARS-CoV infection. In a Syrian Golden hamster wildtype SARS-CoV-2 infection model, treatment of VIR-7831 significantly reduced total viral load compared with the control mAb, without a significant impact on bodyweight change [98]. This study provides a valuable insight into expanding multiple functional mAbs.

### 3.2. Antibody Cocktail

Due to the rapid mutation of viruses, the efficacy of mAbs is influenced. Thus, a cocktail of two or multiple antibodies may confer a powerful neutralizing effect against viral variants. Antibody cocktails for SARS-CoV-2 such as a cocktail of Tixagevimab plus Cilgavimab have been issued by FDA for emergency use against COVID-19 infection [99]. This cocktail also showed a neutralizing effect on Delta and Omicron variants [100,101].

There are many ongoing and finished studies for the efficacy evaluation of antibody cocktails. Some new approaches are worthy to specifically point out. One study reported a novel approach using protein structures to guide the design of antibody cocktails. The development of structure-guided antibody cocktails displays some great advantages. Some of these cocktails showed potent neutralizing ability against all the variants such as Alpha, Beta, Gamma, Epsilon, Iota, Kappa, and Delta in mouse and hamster models [102,103]. These cocktails hold promise and the potential to become one of the useful tools to combat viral infection, especially for the infection caused by highly mutated variants.

### 3.3. Engineered Bispecific Monoclonal Antibody

A recent study reported a bispecific single-domain antibody, named bn03. It was designed based on the highly conserved region of RBD of Omicron variants. Interestingly, the bispecific bn03 showed a significantly higher naturalization efficacy compared with the cocktail combination of n3113v and n3130v. The EM-crystal structure analysis of Omicron (S-bn03) showed that it includes two arms, and both arms can simultaneously bind with the single RBD. The synergistic effect conferred by the two arms of bn03 increased the binding affinity of antibodies to viruses. Therefore, the neutralization effect of bn03 was increased. In addition, due to its small size, bn03 was able to bind to the most conserved cryptic epitopes in the deeper binding site. Moreover, the team verified that inhalation is the best method for bn03 delivery compared with other delivery paths [86]. Although there are some limitations such as limited sample numbers, this well-designed study still provides us positive inspiration for using a similar approach to engineer antibodies for virus treatment.

In another study, two different bispecific antibodies were generated using a distinct design and were compared with their parental antibodies and their cocktails. One is tetravalent bispecific antibody 14-H-06, and another is a bivalent bispecific antibody 14-crs-06. Both in vitro and in vivo studies demonstrated that 14-H-06 coffered a significantly higher neutralization potency compared with 14-crs-06 (Figure 2E). Both bispecific (tetravalent and bivalent) antibodies showed greater efficacy compared with their parent antibodies or their cocktail [104]. This study illustrates that using an engineered bispecific antibody is a strategy to improve the efficacy of the parental antibody. 

## 4. Peptide-Based Inhibitor

Small peptides can be designed and used as inhibitors to prevent the infection of viruses and other infectious pathogens [105,106]. For example, the N-terminal helix of the hACE2 contains the amino acids that mediated the interaction of hACE2 with the virus. This sequence has been used as a model to generate peptides that mimic the hACE2 (Figure 3F) [107]. The de novo design of miniprotein or peptides targeting the RBD binding site showed both increased binding affinity and enhanced stability. In the same study, the computationally generated scaffolds were also designed to stay around the ACE2 helix and interact with the virus RBD. In such a way, the scaffolds can block the direct interaction between ACE2 and virus RBD. The stability of the binding was further confirmed by the Cryo-EM structure [108]. Using the above-mentioned methods to design small peptides or miniproteins is another strategy to develop pharmaceutical medicines for viral infection (Figure 3F).

## 5. Chemical Compounds

Chemical compounds can be classified as repurposing compounds [109] and de novo designed compounds [110]. The repurposed compounds are initially developed for the treatment of other diseases, including proved and unproved compounds [111]. Based on the origin and source, compounds can be categorized as synthetic compounds and natural product-derived compounds [112]. Their therapeutic function is conferred through several mechanisms such as (1) blocking and inhibiting the interaction of virus and host [113], (2) interrupting the signaling pathway of disease pathogenesis [114], (3) inhibiting the virus evasion, replication, translation, and proliferation [115], and (4) modulating the inflammation and facilitating host immune response [116].

**Figure 4:**
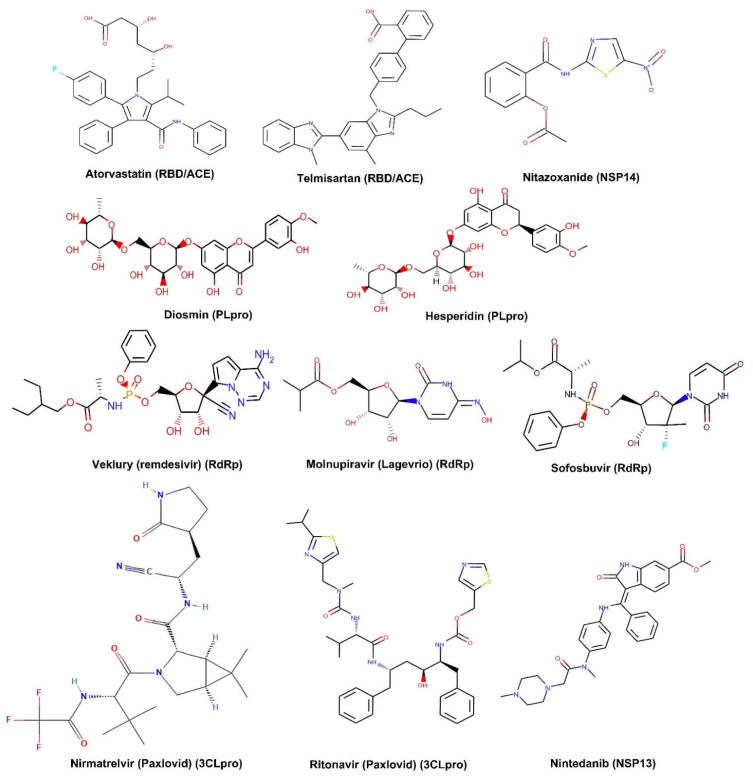
Small molecules targeting spike protein and nonstructural proteins of SARS-CoV-2. Abbreviations: ACE: angiotensin-converting enzyme 2; NSP: nonstructural protein; PLpro: papain-like protease; RBD: receptor-binding domain; RdRp: RNA dependent RNA polymerase; 3CLpro: 3C-like protease.

The targets of small-molecule binding sites are mainly focused on viral proteins. The target selection is based on protein functions, which are illustrated in detail in the first section of this context. These functional proteins include both the SPs and NSPs of the virus. For instance, a small molecule that targets host ACE2 or TMPRSS2 protease can block viral binding, thus the entry of viruses is reduced and blocked [117,118]. Similarly, small molecules also can be designed to bind with the viral S protein, the major protein mediating virus entry into the host cells [119]. Targeting SARS-Cov-2 SPs such as the S protein and RBD has drawn intensive attention in the field. Small molecules that target RdRp such as Remdesivir show the capability of inhibiting the virus RNA replication (Figure 3G) [120]. Molecules such as Lopinavir/ritonavir serve as protease blockage [121]. Another molecule favipiravir shows a potent polymerase inhibitive function [122]. In summary, most currently available small molecules target the S protein and RBD for SPs [123]. For the NSPs of SARS-CoV-2, most small molecules mainly bind to 3CLpro, PLpro, Helicase, and RdRp [124,125,126,127]. In addition, some small molecules such as dexamethasone and chloroquine showed anti-inflammation function during SARS-CoV-2 infection [128]. Using chloroquine or hydroxychloroquine in combination with azithromycin and/or others can show a synergistic effect on viral infection [129]. However, more clinical validation is required [130,131]. Examples of the most investigated small molecules and their targets are summarized in a table (Table 1). Their molecule structures are displayed in a graph (Figure 4).

## 6. CRISPR Cas Technology

CRISPR technology is an attractive gene-editing platform. Currently, several investigations attempt to use the CRISPR system to detect SARS-CoV-2 as a diagnostic tool. Importantly, there are limited data regarding it as a therapeutic option for COVID-19. However, CRISPR technology has been proposed as attractive machinery for fighting against virus infection [132,133,134,135]. A CRISPR/Cas13 system was designed and reported, which showed a valuable tool to precisely edit the RNA [136]. The same team also developed a CRISPR-Cas13-based SHERLOCK (specific high-sensitivity enzymatic reporter UnLOCKing) technique system. This system can be used to detect the COVID-19 virus [137,138,139]. Recently, a study on CRISPR/Cas13-based therapeutic function by targeting RdRp against SARS-CoV-2 infection was explored. The results clearly illustrate the function of the CRISPR technique in the detection of the RdRp of SARS-CoV-2. However, it is still challenging to demonstrate the treatment function [140]. It is worth further exploring its therapeutic potential. In summary, CRISPR is a powerful gene-editing tool, and further investigation is required to apply this technique in viral therapy.

In addition to antiviral treatment, it is critical to give treatment according to the disease’s symptoms. In patients, two clusters of clinical symptoms are commonly associated with SARS-CoV-2 infection, including (1) ageusia, anosmia, and fever and (2) shortness of breath, coughing, and chest pain [141]. A cytokine storm is associated with a high rate of COVID-19 mortality by causing hyperinflammation [142,143]. Multiple strategies have been applied to treat COVID-19 symptoms to manage this disease [144,145]. For example, high levels of IL-6 and IL-8 were significantly associated with a higher death rate in COVID-19 patients [146]. Therefore, anti-inflammation treatment should be first considered. IL-6-targeted therapeutic options including anti-IL-6 mAb Siltuximab [147] and anti-IL-6 receptor mAbs Tocilizumab and Sarilumab [148,149] have been applied to inhibit the cytokine storm-induced hyperinflammation. In addition, small molecules, such as furosemide with inhibitory function for IL-6 and TNF-α [150,151], can also be applied for treatment. For the COVID-19-infected patients with diabetes, cancers, or other chronic diseases with other complicated medical situations, the treatment strategy should be carefully selected.

## 7. Conclusions

Upon the emerging and rapid spreading of virus infections, the development of an effective vaccine on time is critical to slow down the contagious disease. However, technology-based strategies may not be available, such as vaccine development and exploration of the delivery system. In addition, in vitro and in vivo preclinical studies and clinical trials are necessary for evaluating the safety of a new vaccine. All these procedures are time-consuming. Thus, the spread of initial infection before the development of an effective vaccine or therapeutic option is unavoidable. Therefore, the timely exploration of alternative treatment strategies is essential.

Notably, the exploration of a treatment strategy for any viral disease needs a better understanding of viral protein structure and function, the mechanism of infection pathogenesis, and the signaling pathways of the host immune responses. The conformational plasticity and function of the S protein play a fundamental role in virus pathogenesis. Viral mutations could cause a conformational change in the S protein and influence the binding affinity between the viral RBD and host ACE2, consequently resulting in the change in stability of the RBD–ACE2 complex. Some mutations contribute to the escape of antibody neutralization. Thus, the effectiveness of antibodies and vaccination is influenced. The glycosylation of the S protein is also closely associated with the viral virulence and escape of host immune surveillance. 

By uncovering the viral protein structures and functions, we can develop effective vaccines, antibodies, and small molecules against viral infection at different stages. Currently, antiviral infection medicines remain unmet. Different approaches can be applied to the development of preventive and therapeutic strategies, such as monoclonal antibodies, peptide-based inhibitors, bioactive molecules, and CRISPR technology-mediated therapy. Advancing our techniques helps develop new tools and medicines against viral infection promptly, which is also critical to prevent a future outbreak of pandemic diseases. Overall, to prepare well for an unpredictable pandemic in the future, investigation into both virus vaccines and antiviral medicines is required.

## Figures and Tables

**Table 1:** Chemical compounds targeting spike protein and nonstructural proteins for SARS-CoV-2 in clinical studies.

Protein	Durg Target	Chemical Compound	Clinical Number	Status
Spike	RBD/ACE2 blocker	Atorvastatin	NCT04380402	Phase 2
		Telmisartan	NCT04355936	Completed
		Chloroquine	NCT04303507	N/A
NSP3	Papain-like protease	Diosmin/Hesperidin (ACE2)	NCT04452799	Early Phase 1
NSP5	3CLpro	Nirmatrelvir (Paxlovid)	NCT04960202 NCT05047601 NCT05011513	FDA approved
		Ritonavir (Paxlovid)		
NSP12	RdRp	Veklury (remdesivir)	NCT04292730	FDA approved
		Molnupiravir (Lagevrio)	NCT04575597	FDA approved for EUA
		Sofosbuvir	NCT04460443	Phase 2
NSP13	Helicase	Nintedanib	NCT04541680	Phase 3
NSP14	Endoribonuclease (3′-5′)	Nitazoxanide	NCT04561219	Phase 2
NSP15	Uridine-specific endoribonuclease	Paritaprevir	NCT02504099	N/A
NSP16	Methyltransferase	Bemcentinib	NCT04890509	Phase 2

Abbreviations: ACE: angiotensin-converting enzyme 2; N/A: not applicable; NSP: nonstructural protein; PLpro: papain-like protease; RBD: receptor-binding domain; RdRp: RNA dependent RNA polymerase; 3CLpro: 3C-like protease.

## Data Availability

All data supporting the report are included in the paper.

## Abbreviation

ACE2: angiotensin-converting enzyme 2; CD4: cluster of differentiation 4; CRISPRs: Clustered regularly interspaced short palindromic repeats; Cas13: CRISPR associated protein 13; CRISPR/Cas: clustered regularly interspaced short palindromic repeats–associated nucleases; E: envelope protein; ER: Endoplasmic reticulum; hACE2: Human angiotensin-converting enzyme 2; IgG: Immunoglobulin G; IL: interleukin; IRF3: Interferon regulatory transcription factor 3; NLRP3: Nod-like receptor protein 3; INF: Type I interferon; M: Membrane protein; mAb: monoclonal antibody; MAM: mitochondria-associated ER membranes; MAVS: Mitochondrial antiviral signaling; NMP: Nucleotidyl-monophosphate; N: nucleocapsid protein; NSP: nonstructural protein; NF-ĸB: nuclear factor kappa B; ORFs: Open reading frames; PALS1: Protein associated with Lin-Seven 1; PLpro: Papain-like protease; RBD: receptor-binding domain; RIG-I: Retinoic acid-inducible gene I; RIPK1: Receptor-interacting serine/threonine-protein kinase 1; RdRp: RNA dependent RNA polymerase; SHERLOCK: Specific high-sensitivity enzymatic reporter UnLOCKing; SUMO: Small ubiquitin-like modifier; S: Spike protein; SARS-CoV-1: Severe acute respiratory syndrome Coronavirus-1; SARS-CoV-2: Severe acute respiratory syndrome Coronavirus-2; semiSWEET: sugars will eventually be exported transporters (SWEETs); 3CLpro: Chymotrypsin-like protease; TMPRSS2: Transmembrane serine protease 2; STAT1: Signal transducer and activator of transcription 1; STAT2: Signal transducer and activator of transcription 2; TNF-α: Tumor necrosis factor alpha; UTR: untranslated region; VH: Heavy chain variable region; VL: Light chain variable region.
